# Do Children Carry the Weight of Divorce?

**DOI:** 10.1007/s13524-019-00784-4

**Published:** 2019-06-11

**Authors:** Alice Goisis, Berkay Özcan, Philippe Van Kerm

**Affiliations:** 1 Department of Social Policy, London School of Economics, Houghton Street, London, WC2A 2AE UK; 2 Max Plank Institute for Demographic Research, Rostock, Germany; 3 Centre for Longitudinal Studies, Department of Social Science, University College London, London, UK; 4 Luxembourg Institute of Socio-Economic Research and University of Luxembourg, Esch/Alzette, Luxembourg

**Keywords:** Divorce, Children, BMI, Obesity, Event Study

## Abstract

Relatively few studies have examined the physical health of children who experience parental separation. The few studies on this topic have largely focused on the United States and have used cross-sectional designs. Our study investigates the relationship between parental separation and children’s body mass index (BMI) and overweight/obesity risk using the UK Millennium Cohort Study. Treating parental separation as a process, we analyze variations in children’s physical health before and after the date of their parents’ separation in order to capture potential anticipation, adaptation, delayed, or cumulative effects. We estimate fixed-effects models to account for the potential correlation between children’s physical health and unobserved factors associated with parental separation, such as socioeconomic background and other time-invariant parental characteristics. We find no evidence of statistically significant anticipation effects in the build-up to parental separation or of statistically significant changes in children’s physical health immediately after separation. However, our results show that in the longer term, the BMI of children whose parents separate significantly deviates from the BMI of children from intact families. Furthermore, this association is especially strong for separations that occur when children are under age 6.

**Electronic supplementary material:**

The online version of this article (10.1007/s13524-019-00784-4) contains supplementary material, which is available to authorized users.

## Introduction

Scholars have long studied the effects of parental breakup on children’s lives and well-being. A large body of literature has analyzed the relationship between family dissolution and child outcomes, such as cognitive skills, educational outcomes, and emotional and psychological well-being (for recent reviews, see Amato 2010; Bernardi et al. 2013; McLanahan et al. 2013). Only a few studies have examined the relationship between family dissolution and children’s physical health (Augustine and Kimbro 2017; Biehl et al. 2014; Bzostek and Beck 2011; McConley et al. 2011; Schmeer 2012; Yannakoulia et al. 2008). Yet, parental separation may negatively affect children’s physical health by changing the quality and the quantity of resources available in the family or by imposing stress and altering children’s environment. In turn, poor health in childhood might have long-lasting effects on various health and social outcomes in adulthood (e.g., Case et al. 2003; Reilly and Kelly 2011).^1^

The findings of these few studies are mixed, with some showing no association between family breakup and children’s physical health (e.g., Augustine and Kimbro 2013; McConley 2011), and others showing a negative association (Bzostek and Beck 2011; Schmeer 2012; Yannakoulia et al. 2008). Given that almost all these studies relied on cross-sectional data, their results may be influenced by unobserved family characteristics that affected both the risk of separation and children’s physical development. We know of only one study that controlled for such unobserved time-invariant confounders: drawing on the Fragile Families longitudinal data set, Schmeer (2012) used within-child (fixed-effects) models that controlled for such unobserved time-invariant confounders. Like most other cross-sectional studies, this research focused on the United States. To our knowledge, no study has examined the effects of parental separation on children’s physical health using longitudinal and representative data from outside the U.S. context.

This article has three original features. First, we provide new evidence on the relationship between parental separation and children’s physical health in the United Kingdom using the Millennium Cohort Study (MCS), a nationally representative longitudinal data set of children born around 2001 and followed until age 14. Second, unlike earlier studies, we rely on child fixed-effects models, which allow us to control for observed and unobserved time-constant family characteristics (e.g., genetic factors, parental personality, ethnicity, and parental education) and to address issues of selective exposure to parental separation. Third, unlike in many earlier studies on parental separation and children’s well-being, our modelling strategy treats separation as a process rather than as a single event. Conceptualizing parental breakup as a process that develops slowly, rather than as a discrete event, allows us to include the family conflict period that often precedes parental separation as part of the dissolution process and hence as part of the separation effect that may unfold before the actual separation (for a similar discussion on cognitive and noncognitive skills, see Kim 2011; for a discussion on mental health, see Strohschein 2005; for a general discussion, see Amato 2010). We also examine how parental separation can affect children’s health over time. This aspect is critical because unlike other child outcomes, such as emotional well-being, the effects of parental separation on children’s physical health may not be immediately observable but could emerge with a time lag. To investigate these pre-, post-, and longer-term effects, we exploit the longitudinal nature of the MCS and detailed information about the precise year and month of parental separation. We also show how the effects of separation vary depending on whether children were younger or older when their parents separated.

To measure children’s physical health, we focus on children’s body mass index (BMI) and on overweight and obesity. Recent reviews have concluded that a high BMI is a good indicator of excess adiposity and is a predictor of health problems, especially among relatively heavy children (e.g., Freedman and Sherry 2009). Being overweight or obese in childhood is related to a wide range of health problems in both childhood and adulthood (Daniels 2006).

Our results show that compared with children in intact families, children who experience a parental separation between the ages of 9 months and 11 years have larger increases in BMI and higher risks of overweight and obesity. The effects become stronger with the length of time since separation. The effect sizes in our estimations are larger (in absolute terms) than those typically found in school-based child obesity prevention programs and are comparable with those found in home-based and community-based programs that target child overweight and obesity (see meta-analytical reviews in Stice et al. 2006; Wang et al. 2013). Our results reinforce previous findings that what happens in the home and family environment is crucially important for child overweight and obesity.

## Background

There are many paths through which a parental breakup may affect a child’s net nutrition intake (i.e., nutrition intake minus energy spent), which ultimately determines the child’s BMI and physical health. These paths, which can be grouped into direct and indirect mechanisms, may operate differently before and in the short and longer term after parental separation and may depend on the child’s age when the parents separate.

Direct mechanisms link parental breakup to changes in the amount and type of nutrition children receive and the amount of energy they expend. *Resource theories* are mainly concerned with the household resources available to help children develop physically (e.g., McLanahan and Sandefur 1994). The child’s food intake and energy expended are directly related to the resources available in the home environment, which are provided mainly by the parents.

It is important to distinguish between economic resources and non-economic resources, such as the amount of parental time and attention available to children. The relative importance of these two types of resources may vary over childhood and across the stages of the separation process, although there can be a considerable interplay between them (e.g., Bzostek and Beck 2011; Case and Paxson 2001; Schmeer 2012). After separation, the economic resources available to the mother and the children may decline (see Amato 2010; McLanahan and Sandefur 2009). The implications of this reduction in resources may include changes in the nutrition intake. For example, the mother may have less money to buy fresh vegetables or less time to prepare healthy food at home because of her increased market work. The pressure on the mother’s time and economic resources may affect the energy expended by the children as well. There may also be less money in the household to pay for extracurricular activities (e.g., sports). Compared with married parents, divorced parents may have less time to establish and observe routines and eating schedules, and they may be more likely to serve their children restaurant food, ready meals, or processed food. However, the changes in the weight of the children might be observed only after they have been exposed to a post-separation family environment with reduced resources for a sufficiently long period: the effects of parental separation on children’s BMI might be visible only in the longer term.

A reduction in parental resources, changes in the amount of attention parents devote to their children’s health, and physical development may occur even before separation*,* especially during the conflict period. Recent research in economics and sociology has found that many married women with children increase their labor supply when marital conflict and the risk of divorce increase (Bargain et al. 2012; Genadek et al. 2007; Papps 2006; see the literature reviewed by Özcan and Breen 2012). Consequently, the amount of time and monetary resources available to children may change *before* separation occurs. Thus, it is important to consider the pre-separation period as part of the separation effect (see also Pronzato and Aassve 2019).

Even if the economic resources available to children remain largely unaffected, parental separation may affect children’s health through indirect mechanisms. *Family instability and stress theories* (e.g., George 1993) suggest that even if the resources stay the same before and after the parental separation, changes in family structure are intrinsically harmful for children because of changes in roles, routines, parenting quality, and parental contact (e.g., Lee and McLanahan 2015; Osborne and McLanahan 2007; Thomson and McLanahan 2012).^2^ Changes in family structure create stress for both parents and require children to adjust to the new environment (Cavanagh and Huston 2006; Wu and Martinson 1993). The adjustment may be more stressful if children change their residence or move to a different neighborhood after parental break up (Astone and McLanahan 1994).^3^ Stress and emotional problems related to parental separation may manifest themselves as changes in the eating behavior of children and may cause disruption in their routines. If children respond to changes in the family environment by modifying their eating habits, sleep routines, or physical activity levels, then they may consequentially lose or gain weight. Previous research in the United Kingdom has found that children who had better emotional self-regulation were less likely to be obese at later ages and that emotional self-regulation is associated with regular bedtime, mealtime, and TV time routines (e.g., Anderson et al. 2017). Although the evidence is less consistent, psychological stress was found to be associated with obesity in early childhood also through biological pathways, such as changes in hormonal levels (e.g., Miller and Lumeng 2018). Children may show signs of stress and emotional adjustment problems both before and after parental separation for different reasons. Thus, the extent to which a child’s physical development is affected by a parental separation might depend on the timing (in the pre- or post-separation period) and the pace at which the socioemotional problems generated by the parental separation process influence the child’s weight gain or loss.

Even if the available economic or time resources remain constant, a mother’s emotional response to separation may also affect her children’s eating patterns because of changes in social control in the home environment (e.g., Wynn and Bowering 1990). Changes in mother’s relationship status are associated with high maternal stress and harsh(er) parenting practices (e.g., Cooper et al. 2009). In addition, divorced mothers are more likely than married mothers to overfeed their children (e.g., Bowering and Wynn 1986), and parenting styles may influence children’s dietary behavior, such as eating vegetables or having breakfast regularly (e.g., Pearson et al. 2010). If changes in parenting styles are indeed associated with changes in family context, then a separation could have adverse effects on children’s eating behavior, sleep and TV routines, and so forth through changes in parenting styles and practices. Because it might take time for routines and parenting practices to change, the effects of parental separation on children’s BMI through indirect mechanisms might be detectable only in the longer term.

All these mechanisms imply that the experience of parental separation overall has adverse effects on children’s physical development, including potential weight loss and gain. However, whereas children in low-income countries marked by food insecurity tend to experience stunting and malnutrition (in the context of family structure, see Bronte-Tinkew and DeJong 2004; Desai 1992), we would expect to find in the U.K. context that the main negative effect of parental separation on children’s health would take the form of weight gain. Furthermore, dietary disorders that lead to underweight very rarely appear before the teenage years (Rosen 2003). We expect that in the United Kingdom, on average, family instability is more likely to move children toward the right tail of the BMI distribution and thus toward an increased risk of overweight and obesity.

Previous studies have noted that in theory, the risks associated with parental separation differ for different child outcomes and may vary depending on the child’s age when the parents separate. However, empirical results on the effects of the child’s age at parental separation on outcomes other than physical health have been mixed (Amato 2001; Härkönen et al. 2017). A few of these studies have suggested that a parental breakup that occurs during early childhood (before ages 5–6) or the mid-teenage years is particularly detrimental to social and educational outcomes (e.g., Cavanagh and Huston 2006; Cherlin et al. 1998; King 2002). As the need for net nutrition varies with age, the mechanisms that link child physical health and parental separation may affect a younger child differently than an older child. The effect of the child’s age at parental separation is also associated with both the length of the child’s exposure to the post-separation period (until the outcomes are observed) and with the specific age when the child experienced the pre-separation conflict period. We, therefore, need to analyze the pre- and the post-separation periods separately for younger and older children.

Finally, previous studies on family structure changes have increasingly stressed that those who experience a parental separation are at a greater risk of experiencing subsequent family transitions, generating complex family trajectories, which are likely to be detrimental for children’s socioemotional behavior (Osborne and McLanahan 2007) and their cognitive skills (Fomby and Cherlin 2007). Children’s well-being is affected independently by both the cumulative nature of experiencing more than one transition and by the destination of these transitions in the form of living in stepfamilies (Sweeney 2010), involving complex relationships with stepsiblings and half-siblings (Fomby et al. 2016). Thus, part of the post-separation effects could plausibly be driven by a subset of children who experience further family transitions. These subsequent family transitions may induce further stress generated by instability, as both family resources theory and family instability and stress theory would predict.

In short, many potential mechanisms link parental separation to children’s physical health. Although testing each of these mechanisms is beyond the scope of this study, there is no ambiguity in the predicted direction of the association: all these theoretical mechanisms predict that parental separation will lead to a worsening of children’s physical health and to an increase in children’s BMI and risk of overweight and obesity. Different mechanisms might play out at different speeds and during different stages. Thus, the overall effects of divorce on children’s BMI could emerge from processes that operate both before and after separation. These effects might become visible only in the longer term after separation because it could take time for family routines, eating habits, and parenting practices to change, but also because children’s weight gain might happen gradually. It is therefore critical that we adopt a process-oriented approach, as Amato (2010) and Kim (2011) suggested.

## Data and Methods

### Data and Sample Construction

We use data from the Millennium Cohort Study (MCS), a U.K. representative longitudinal cohort study that follows the lives of a sample of children born between September 2000 and January 2002. The initial data were collected when children were approximately 9 months old. Five subsequent survey waves were collected when children were approximately ages 3, 5, 7, 11, and 14. At each round, the MCS collected a range of data about the family environment and child health and development. We are therefore able to examine whether parental separation affects the health outcomes during the childhood years of a large and representative sample of a recent birth cohort in the United Kingdom. To focus on the period before the onset of adolescence, we do not consider Wave 6, which was collected when the cohort members were approximately age 14.

The baseline MCS sample includes 19,244 children. Because our focus is on children at risk of experiencing parental separation, we exclude 4,887 children whose parents were not married or cohabiting at Wave 1. Of the remaining 14,357 children, 7,751 were observed over Waves 1–5, and complete information on each variable related to their health and family structure was collected at each wave. From this sample, we eliminate the children for whom a natural parent was not the main interviewee at each wave (74 cases) or who experienced the death of at least one parent (103 cases).^4^ This process results in a balanced sample of 7,574 children that forms our main estimation sample.

Attrition is a concern because our estimation sample includes approximately 50 % of the children observed in the pool of children at risk of parental separation at Wave 1.^5^ Attrition driven by observable factors is accounted for by sample weight adjustments (and/or the inclusion of appropriate control variables in the regression models). If, however, attrition is correlated with parental separation and the severity of the impact of the separation on the child’s health, consequences can be twofold: (1) children experiencing separation are underrepresented in the balanced sample, and (2) the measured effect of separation on child health may be biased. The first consequence should not bias our main regression estimates, but it may reduce statistical precision and thus lead to increased confidence intervals. The second consequence is potentially more serious. However, we expect that it would bias our estimates downward given that the children who experienced the most disruptive separations are the ones who are most likely to have disappeared from our sample and to have experienced large health impacts. Attrition is therefore likely to make our estimates more conservative. Thus, these estimates should be interpreted as reflecting lower bounds.^6^

### Key Variables

Our variables of interest are measures of child physical health—specifically, measures of BMI, obesity, and overweight—collected at Waves 2, 3, 4, and 5 (when children were approximately ages 3, 5, 7, and 11) and information about the experience and timing of parental separation between the ages of 9 months and 11 years. These four observations amount to a total of 30,296 child-wave observations. The theoretical arguments outlined in our earlier discussion on background do not distinguish between cohabitation and marriage. We consider all union dissolutions because cohabitations have been found to be more committed and marriage-like in the United Kingdom than in the United States (e.g., Kiernan et al. 2011). Moreover, we do not distinguish between children living with their father or with their mother after separation because of sample size limitations; more than 95 % of the respondents were living with their mother post-separation.

#### Measuring Children’s Physical Health

Our first key aim is to design a physical health measure that is reliable and comparable for children of both genders and different ages.

We rely on BMI, which combines height and weight (kg/m^2^) and is considered to be a reliable measure of child adiposity and a good predictor of health risks (Freedman and Sherry 2009; Pietrobelli et al. 1998). A clear advantage of using this child health measure is that it was obtained by a trained interviewer in the MCS—that is, BMI was not based on reports by the parent, which could be subject to error and bias. At each wave, children were weighed without shoes or outdoor clothing using Tanita HD-305 scales (Tanita UK Ltd, Middlesex, UK), and their weights were recorded in kilograms to one decimal place. Heights were obtained using the Leicester Height Measure Stadiometer (Seca Ltd, Birmingham, UK) and recorded to the nearest millimeter.

Because BMI distributions evolve rapidly during childhood and differently for boys and girls, we need to make age and gender adjustments (Cole et al. 2000). This pattern is illustrated in the online appendix, Fig. A1. It is necessary to account for biological changes in BMI as children grow older. We can use unadjusted BMI as a dependent variable and include controls for age and gender in the regression equations to partial out growth effects. Such an approach may rely on undesirably strong parametric assumptions about the relationship between age and BMI (e.g., a quadratic relationship). Therefore, we also adopt an age- and gender-adjusted measure that converts BMI measurements into *z* scores based on the 1990 British Growth Reference for age and gender (Cole et al. 1998; Vidmar et al. 2004). Compared with the first measure, this BMI indicator eliminates age and gender differences more thoroughly and with fewer assumptions. By construction, the *z* scores follow a Gaussian distribution within each age and gender. The standardization from the British Growth Reference is applied for each age and gender.^7^ Fig. A2 in the online appendix shows the distribution of the standardized measure in our data: gender and age differences are largely eliminated. We use the resulting BMI *z* scores as the second dependent variable in our analyses.^8^

Although changes in standardized BMI measures are easily interpretable, they may mask changes at the tails of the distribution (see in Fig. A1 how the 95th percentile of BMI increases after age 7). Therefore, we adopt as an additional measure a categorical variable indicating whether the child is overweight or obese. We combine overweight and obesity status because both conditions have been found to be associated with worse health and social outcomes in childhood and adulthood. Overweight and obesity are defined using the International Obesity Taskforce (IOTF) BMI cut points, which are age- and gender-specific.

In sum, throughout the analyses, we use three measures of child health, the last two of which are age- and gender-adjusted: (1) BMI, (2) BMI *z* scores, and (3) an overweight/obesity binary indicator.

#### Measuring the Time From and to Separation

Our second key aim is to create a measure of parental separation and the time to and from the separation.

The interviewed parent who experienced a separation between any two MCS waves was asked to report the month and year when the separation occurred. The question captures the time when the biological parents of the cohort member stopped living together, regardless of whether they were married or cohabiting before separation. By combining this information with the interview date, we calculate the number of months that elapsed between the separation and the moment when the child’s health was measured.^9^ This step is important because the time elapsed between two interviews could be relatively long and varies considerably between children. Combining the date of separation with the date of interview (rather than with the theoretical wave age) is necessary because the interviews were not conducted when the children were exactly ages 3, 5, 7, and 11 (the cohort members were born between 2000 and 2002). In each wave, age of the youngest and the oldest respondent differs by up to 20 months.

Table 1 shows the percentage of separations that occurred between each wave. In total, 1,573 children (approximately 20 % of our sample) experienced a separation between Waves 1 and 5. The largest share of the separations took place between Waves 4 and 5, primarily because roughly 48 months elapsed between Waves 4 and 5, compared with only roughly 24 months elapsing between the other waves. Note that the number of separations declined gradually over time as a natural consequence of the reduction in the number of children still living in intact families (and who were at risk of experiencing separation) in our sample.

**Table 1:** Number and percentage of separations that occur between waves

	*N*	%
No Separation	6,001	79.23
Wave 1–Wave 2	375	4.95
Wave 2–Wave 3	350	4.62
Wave 3–Wave 4	310	4.09
Wave 4–Wave 5	538	7.10
Number of Children	7,574	

*Note:* Row percentages do not add up to 100 % because of rounding.

Because weight change is a process that occurs gradually, we calculate retrospectively the time between any BMI measurement in the data and *past* or *future* parental separation observed within the period covered by the MCS. As we explain shortly, this approach allows us to capture potential anticipation effects of an upcoming separation as well as a potential adaptation in the longer run after separation. Using the information on the month/year of separation and the month/year of interview, we create 12-month intervals that capture the pre- and post-separation periods (starting one year before separation).^10^ Table 2 shows the six categories capturing the pre- and the post-separation periods, and the number of observations in each.^11^ We also show the distribution of observations across the separation variable for the children who experienced a separation before or after age 6. Pre-separation observations disproportionately include children who experienced separation after age 6, while (especially the longer term) post-separation observations disproportionately include children who experienced separation before or at age 6.

**Table 2:** Pre- and post-separation observations for cohort children whose parents separate between Waves 1–5, in 12-month intervals

		Pre- and Post-Separation Observations Across Waves				% Pre- and Post-Separation Observations, by Age at Separation^a^		
	%^a^	Wave 2	Wave 3	Wave 4	Wave 5	≤6	>6	Child-Wave Observations
Pre-Separation >12 Months	32.91	1,020	669	373	9	16.76	83.24	2,071
Pre-Separation 12–0 Months	7.84	166	167	160	0	58.01	41.99	493
Post-Separation 0–11 Months	7.91	95	163	131	109	52.01	47.99	498
Post-Separation 12–23 Months	10.62	230	150	165	123	73.95	26.05	668
Post-Separation 24–35 Months	7.63	60	137	148	135	71.88	28.13	480
Post-Separation 36+ Months	33.09	2	287	596	1,197	83.81	16.19	2,082
Total Number of Separations (child-wave observations)	1,573 (6,292)							

^a^Column percentages in the first column may not sum to exactly 100 % because of rounding.

**Fig. 1:**
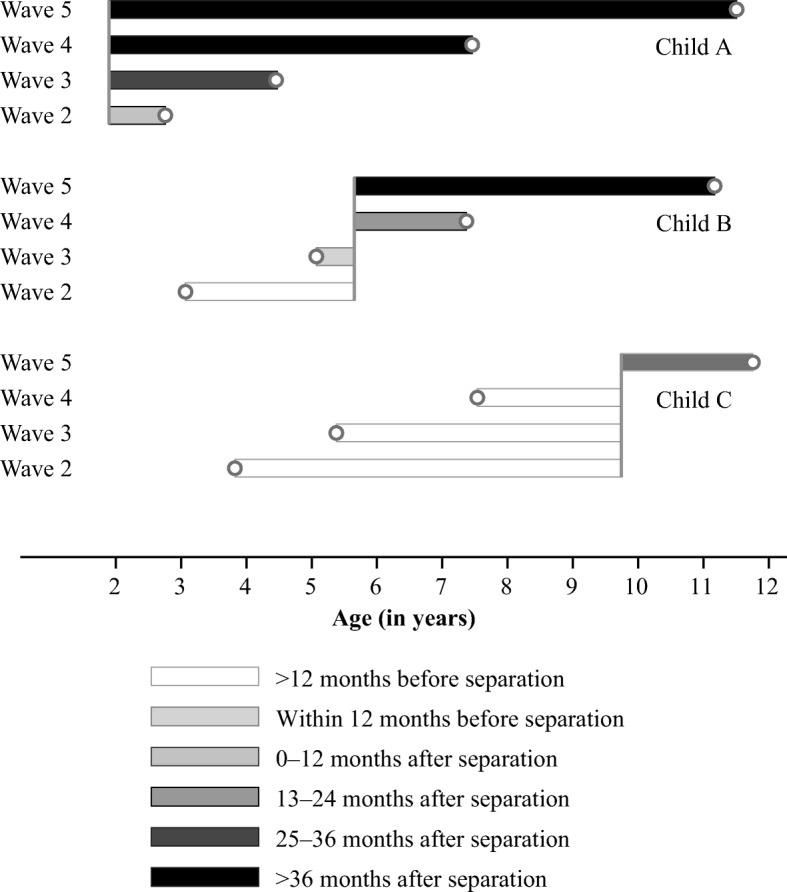
Illustrative examples

Given the MCS design, depending on when their parents separate, different children contribute to differing extents to the estimation of the pre- or the post-separation variables. Figure 1 provides three illustrative examples. Children A, B, and C provide four BMI measurements (at Waves 2, 3, 4, and 5); each observation is represented by a dot. These observations vary by the child’s age at separation and the time since separation. Child A experiences the separation at age 2 (between Waves 1 and 2); all four BMI observations, therefore, take place after the event. The first measurement is taken within 12 months after the separation, the second measurement is taken between 24 and 26 months after separation, and the last two measurements are taken more than 36 months after the separation. Because no measurement is taken before separation takes place, Child A does not contribute to the estimation of the anticipation effects. Child B experiences the separation after she turned 5. The first measurement is taken more than 12 months before the separation (and will be assumed to be unaffected by the separation in our model, as shown in upcoming Eq. (2)).The second measurement is taken within 12 months before the separation (the build-up period), the third measurement is taken between 12 months and 24 months after the separation, and the fourth measurement is taken more than 36 months after the separation. Finally, Child C experiences the separation at approximately age 10. The first three measurements are taken more than 12 months before the separation, and the last measurement is taken between 24 months and 36 months after the separation.

Table 3 displays descriptive statistics for the three outcome variables for the children whose parents remained together between Waves 1 and 5 as well as for their counterparts whose parents separated. MCS longitudinal weights (initial design weights adjusted for attrition between Waves 2 and 5) are used for all calculations. On average, the children who experienced a parental separation between Waves 1 and 5 tended to have higher BMI levels and a higher prevalence of overweight/obesity at all ages, including at the ages *before* separation. This observation is consistent with evidence showing that these children come from disadvantaged families, and thus underlines the importance of applying child fixed-effects models. The model analyzing the association between parental separation and children’s health needs to take into account the unobserved confounders that might influence both the parents’ risk of separation and the children’s BMI and risk of overweight/obesity.

**Table 3:** Descriptive characteristics of the analytical sample and of the children who are dropped from the analyses

Wave	Mean BMI			Mean BMI *z* Score			% Overweight/Obese		
	Children in Intact Families Waves 1–5	Children Experiencing Separation in Waves 1–5, Observed for Entire Period	Children Experiencing Separation After Wave 1, Dropped From Analyses^a^	Children in Intact Families in Waves 1–5	Children Experiencing Separation in Waves 1–5, Observed for Entire Period	Children Experiencing Separation After Wave 1, Dropped From Analyses^a^	Children in Intact Families Waves 1–5	Children Experiencing Separation in Waves 1–5, Observed for Entire Period	Children Experiencing Separation After Wave 1, Dropped From Analyses^a^
2	16.81	16.95	16.84	0.49	0.57	0.50	0.23	0.25	0.24
3	16.25	16.37	16.49	0.40	0.47	0.53	0.19	0.22	0.24
4	16.46	16.65	17.08	0.30	0.38	0.56	0.18	0.19	0.26
5	18.98	19.51	19.53	0.47	0.63	0.60	0.25	0.29	0.32
Number of Children	6,001	1,573	4,289	6,001	1,573	4,289	6,001	1,573	4,289

*Notes:* Balanced sample weighted by Wave 5 longitudinal weights; unbalanced sample weighted by wave-specific longitudinal weight. Number of children: Wave 2 *n* = 1,677; Wave 3 *n* = 1,365; Wave 4 *n* = 598; Wave 5 *n* = 649.

^a^Cases that are not included in the analyses either because of attrition or because of missing values on the variables included in the analyses.

For both groups of children, the average BMI and overweight/obesity levels decreased between Waves 2 and 4 and rose between Waves 4 and 5—a pattern that can be explained by the adiposity rebound that takes place at approximately age 5 or 6 (Whitaker et al. 1998). The children who were not included in the analyses—because of attrition or missing values on the variables included in the analyses—tended to be heavier from Wave 3 onward. This finding supports the assumption that our balanced sample leads to conservative (downwardly biased) estimates of the effects of parental separation.

### Regression Models

The key relationship of interest is between the measures of child body weight and the elapsed time since (or to) the separation at the time of each of those measurements. To ensure that this relationship is not contaminated by how BMI evolves with age and gender, we rely on age- and gender-adjusted measures of BMI and include age controls in our regression models. We adopt fixed-effects models to ensure that our estimates of the effects of separation are not confounded by unobserved time-invariant characteristics of the child or the parents that could influence both the child’s health and the parents’ risk of separation (e.g., genetic factors, socioeconomic background, parents’ education and age at childbirth, and the child’s birth order); see Amato and Anthony (2014) and Wooldridge (2013). Our aim is to capture how much a child’s BMI deviates from his/her average corpulence in the years just before or after the separation (identified by the gray bars in the illustrative examples shown earlier in Fig. 1).

Our specification allows the separation to have a distributed effect both before the actual date of the separation and in the short and the medium term following the separation. We do this by including in the fixed-effects regression model dummy variables that capture whether BMI is measured during the 12 months before the date of separation, or during any of the intervals of 0–11 months, 12–23 months, 24–35 months, or more than 36 months after the separation; see Fig. 1. Specifications of this kind have recently been used to model the effects of marriage on wages (see, e.g., Dougherty 2006) and life satisfaction (Clark et al. 2008): they allow for the estimation of pre- and post-marriage effects on adaptation.

We report three regression model variants. To compare our concepts with those of earlier studies, we first estimate a baseline model that ignores any anticipation or adaptation mechanisms:

1 $$ {h}_{it}=\upbeta\ {Sep}_{it}+{f}_{\uptheta}\left({a}_{it}\right)+{\upalpha}_i+{\mathbf{X}}_{it}\gamma +{e}_{it}, $$

where the dependent variable *h*_*it*_ is one of our measures of child *i* adiposity measured at time *t*; and *Sep*_*it*_ is a dummy variable indicating whether the child experienced a separation at any point in time before time *t*, such that β captures the effect of separation. *f*_θ_(*a*_*it*_) is a parametric function of the age of the child *i* at time *t* (with age expressed in days since birth) that captures the systematic evolution of the adiposity measures of the children in our sample. We assume that the function is quadratic in age—that is, *f*_θ_(*a*) = θ_0_ + θ_1_*a* + θ_2_*a*^2^—to accommodate the nonlinearities in the evolution of the BMI with age.^12^ Crucially, we assume that the shape of the function *f*_θ_ is common to all children and does not differ in shape for the children whose parents separate.^13^ α_*i*_ is a child-specific constant that varies across children but not over time; it shifts the *f*_θ_(*a*_*it*_) function up or down by a constant that is different for each child, and by construction, its average over the sample is 0. We estimate the model parameters in Eq. (1) by the conventional *within transformation* (Wooldridge 2013), such that the child-specific constants α_*i*_ are not actually estimated and are therefore allowed to be freely correlated with any of the (observed or unobserved) time-invariant characteristics of the parent, the family, or the child (including the child’s sex) that may affect both the child’s adiposity and the likelihood of parental separation. In a within-transformation estimation, the presence of such a correlation does not affect the estimation of our key β parameters because the latter are estimated based on the deviation over time from the child-specific BMI trajectory *f*_θ_(*a*_*it*_) + α_*i*_ among the children who experienced a parental separation. **X**_*it*_ is a vector of additional control variables at time *t* for child *i*, which we include only in additional analyses in which we explore potential pathways.

Our second model allows for an anticipation effect of separation in the 12 months before the separation as well as a heterogeneous evolution of the effect after separation. The latter effect can be helpful in distinguishing between resilience and adaptation mechanisms associated with the time elapsed since separation (in which case the effect would vanish in the long run), delayed effects (in which case the effect does not materialize until well after separation), or cumulative effects (in which case the effect of separation accumulates and becomes increasingly important as time goes by):

2 $$ {\displaystyle \begin{array}{c}{h}_{it}={\upbeta}_0d\left(-12\le {s}_{it}<0\right)\\ {}+{\upbeta}_1d\left(0\le {s}_{it}<12\right)+{\upbeta}_2d\left(12\le {s}_{it}<24\right)+{\upbeta}_3d\left(24\le {s}_{it}<36\right)+{\upbeta}_4d\left(36\le {s}_{it}<999\right)\\ {}+{f}_{\uptheta}\left({a}_{it}\right)+{\upalpha}_i+{\mathbf{X}}_{it}\gamma +{e}_{it}.\end{array}} $$

The distributed-effect model is identical to the baseline model except in one respect: it uses five mutually exclusive dummy variables that correspond to the time since/to the separation at the time of interview at wave *t*, instead of merely distinguishing between the pre- and the post-separation interviews. β_0_ picks up any anticipated effect of separation in the 12 months before the actual separation; β_1_ picks up the immediate effect of separation; and β_2_, β_3_, and β_4_ capture the longer-term effects. All dummy variables are set equal to 0 for the children who did not experience a separation in the observation period. We assume that more than 12 months before the separation, there is no observable deviation from the systematic child-specific corpulence given by *f*_θ_(*a*_*it*_) + α_*i*_.^14^

Finally, to address the concern that the child’s age when the parents separate can also be a determinant of the effect of the separation on the child’s health, a third specification allows for different separation effects depending on the child’s age when the separation occurred (e.g., Cherlin et al. 1998; King 2002). For this specification, we use Eq. (2) and distinguish between separations that occurred before or after age 6. We are limited to this broad age range because we cannot observe a child’s BMI for a long period before the separation if the child experienced the separation at a relatively young age or, conversely, at a relatively old age. Thus, we estimate a set of 10 coefficients that capture the impact of separation: 5 for each age-at-separation subgroup. We cannot rule out the possibility that the results about the child’s age at the time of the separation are contaminated by a period effect. Given that the cohort of MCS children in our sample were born between 2000 and 2002, all separations before age 6 occurred before 2006–2008, and all separations after age 6 occurred after 2006–2008. Given the cohort data, we have to assume away any period effect to interpret the interaction between the separation and the age at separation as a pure reflection of the effect of the latter.

## Results

**Fig. 2:**
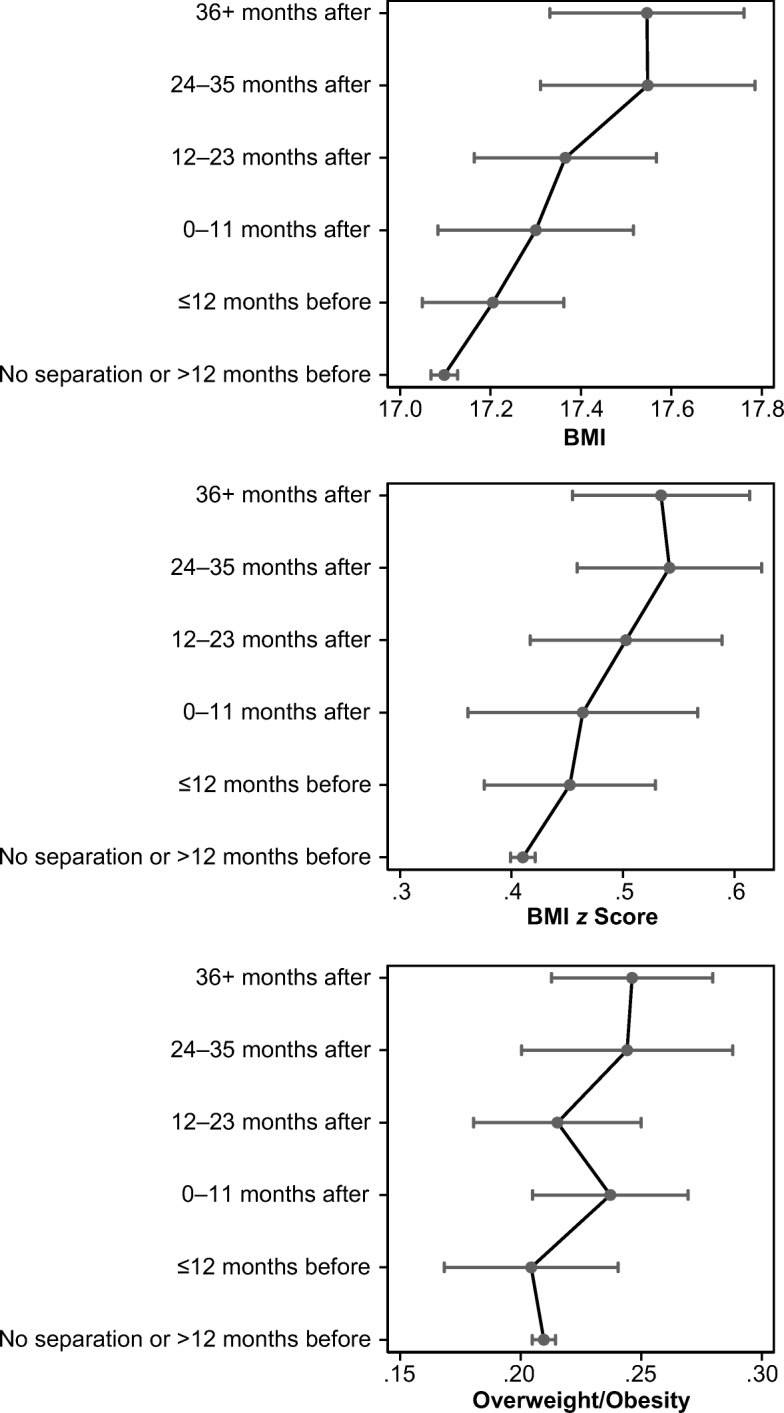
Predicted values of BMI, BMI *z* scores, and overweight/obesity for the time relative to separation. Fixed-effects Model 2, Table 4.

**Fig. 3:**
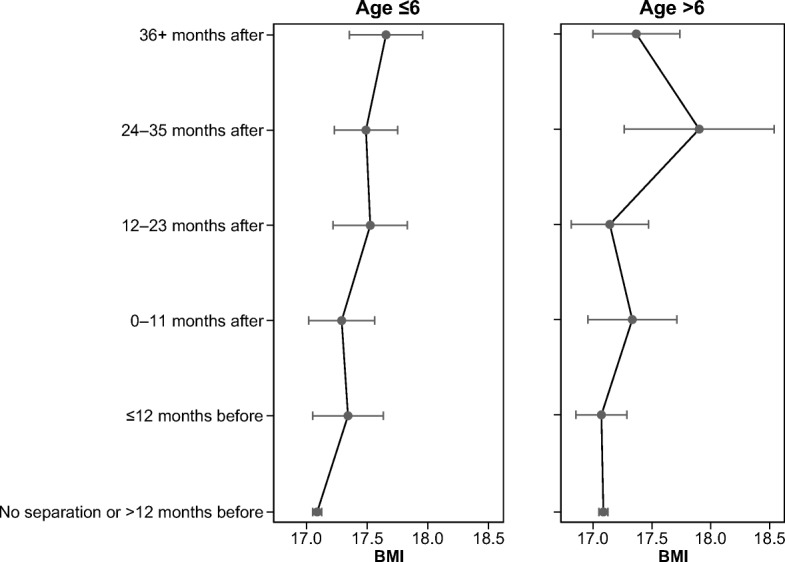
Predicted values of BMI for the time relative to separation by age at separation. Fixed effects from Table 5.

**Fig. 4:**
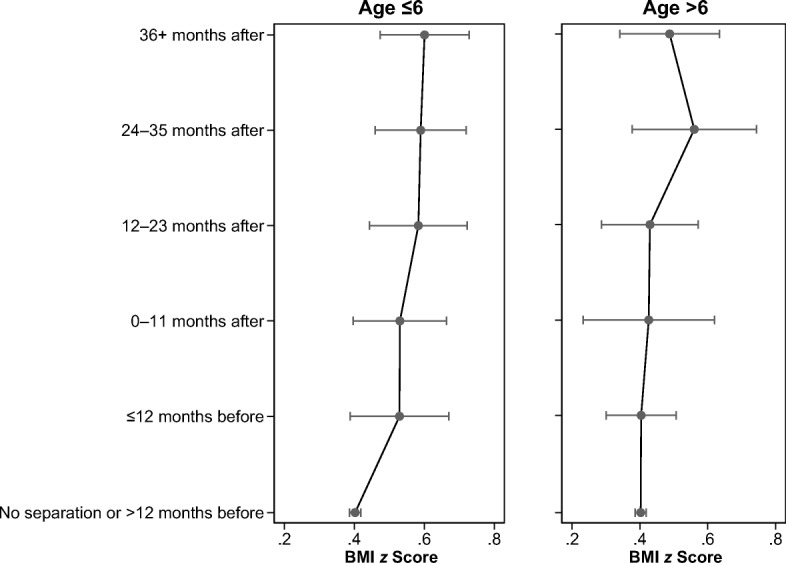
Predicted values of BMI *z* scores for the time relative to separation by age at separation. Fixed effects from Table 5.

**Fig. 5:**
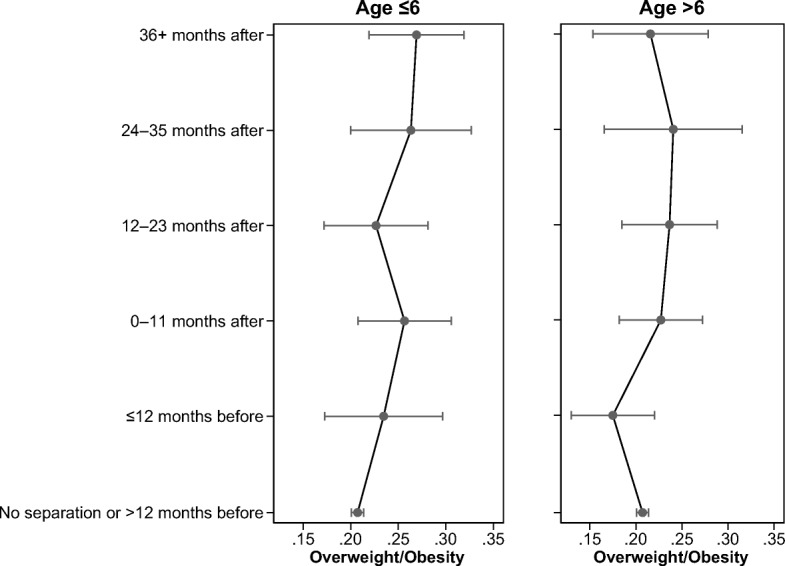
Predicted values of overweight for the time relative to separation by age at separation. Fixed effects from Table 5.

Tables 4 and 5 present our estimation results, and Table A1 in the online appendix reports descriptive statistics of the control variables used in the analyses. Table 4 shows the main model results. Model 1 shows the average before/after association between parental separation and children’s BMI and risk of overweight/obesity, and Model 2 shows the distributed effect of parental separation and children’s adiposity measures by dividing the pre- and post-separation period into 12-month intervals. In all models, standard errors account for clustering at the electoral ward level (the MCS primary sampling unit). The results from Model 2 are the key contribution of this study. As explained earlier, in this and in subsequent specifications, the reference category is composed of BMI measurements taken at least 12 months before separation occurred. Looking at Table 5, we can see whether the results differ according to whether the child experienced separation before or after age 6. Because all the regression models are linear, coefficients are directly interpretable as marginal effects. However, to facilitate the comparison of the magnitude of the separation effect over time, we report in Figs. 2, 3, 4, and 5 the predicted values for each outcome at different values of the separation dummy variables. The predicted values for each observation are calculated from Tables 4 (Model 2) and 5 according to the levels of other covariates averaged across the sample.

**Table 4:** Fixed-effects regression model on BMI, BMI *z* scores, and overweight

	BMI		BMI *z* Scores		Overweight	
	Model 1	Model 2	Model 1	Model 2	Model 1	Model 2
Cohort Member Age in Days (divided by 1,000)	–2.727** (0.054)	–2.739** (0.056)	–0.379** (0.028)	–0.382** (0.028)	–0.115** (0.011)	–0.116** (0.011)
Cohort Member Age in Days Squared (divided by 1,000)	0.000** (0.000)	0.000** (0.000)	0.000** (0.000)	0.000** (0.000)	0.000** (0.000)	0.000** (0.000)
Parents Separated	0.297**		0.085**		0.023^†^	
	(0.079)		(0.030)		(0.014)	
Timing Relative to Separation (ref. = >12 months before)						
0–12 months before		0.108		0.042		–0.005
		(0.089)		(0.042)		(0.020)
0–11 months after		0.202^†^		0.054		0.028
		(0.116)		(0.054)		(0.018)
12–23 months after		0.268*		0.093^†^		0.006
		(0.113)		(0.047)		(0.020)
24–35 months after		0.450**		0.132**		0.035
		(0.131)		(0.046)		(0.024)
36+ months after		0.448**		0.124**		0.037^†^
		(0.124)		(0.046)		(0.019)
Constant	19.005**	19.016**	0.833**	0.835**	0.320**	0.322**
	(0.066)	(0.066)	(0.034)	(0.034)	(0.014)	(0.014)
Number of Observations	30,296		30,296		30,296	
Number of Children	7,574		7,574		7,574	

*Note:* Standard errors, clustered at the primary sampling unit, are provided in parentheses.

^†^*p* < .10; *p < .05; **p < .01

**Table 5:** Fixed-effects regression model on BMI, BMI *z* scores, and overweight for children who experienced separation up to age 6 or after age 6

	BMI	BMI *z* Scores	Overweight
Cohort Member Age in Days (divided by 1,000)	–2.737**	–0.386**	–0.118**
	(0.057)	(0.029)	(0.012)
Cohort Member Age in Days Squared (divided by 1,000)	0.000**	0.000**	0.000**
	(0.000)	(0.000)	(0.000)
Timing Relative to Separation (ref. = >12 months before) by Child Age			
0–12 months before, age ≤6 years	0.254	0.127	0.027
	(0.162)	(0.078)	(0.034)
0–11 months after, age ≤6 years	0.202	0.128^†^	0.049^†^
	(0.150)	(0.074)	(0.027)
12–23 months after, age ≤6 years	0.438*	0.181*	0.019
	(0.172)	(0.078)	(0.031)
24–35 months after, age ≤6 years	0.402**	0.188*	0.056
	(0.148)	(0.073)	(0.035)
36+ months after, age ≤6 years	0.567**	0.199**	0.062*
	(0.172)	(0.073)	(0.029)
0–12 months before, age >6 years	–0.017	0.001	–0.032
	(0.113)	(0.053)	(0.023)
0–11 months after, age >6 years	0.246	0.024	0.020
	(0.195)	(0.099)	(0.024)
12–23 months after, age >6 years	0.055	0.027	0.029
	(0.167)	(0.072)	(0.027)
24–35 months after, age >6 years	0.814*	0.158^†^	0.033
	(0.327)	(0.094)	(0.038)
36+ months after, age >6 years	0.280	0.085	0.009
	(0.193)	(0.076)	(0.033)
Constant	19.005**	0.831**	0.321**
	(0.067)	(0.035)	(0.014)
Number of Observations	30,296	30,296	30,296
Number of Children	7,574	7,574	7,574

*Note:* Standard errors, clustered at the primary sampling unit, are provided in parentheses.

^†^*p* < .10; *p < .05; **p < .01

Model 1 in Table 4 shows that, on average, parental separation is positively and significantly associated with the three BMI measures used in the analyses (at the 1 % confidence level for the continuous BMI measures but only at the 10 % level for overweight/obesity). Model 2 shows how the positive association between the separation and the child’s weight gain is distributed before and after the separation. The coefficient estimates indicate that the child’s BMI tends to increase as the time to separation decreases (pre-separation period) and as the time since separation increases (post-separation period). Figure 2 shows, on average, a monotonic increase in the continuous BMI measures (BMI, BMI *z* scores) starting from one year before separation; the probability of overweight/obesity also increases but does not follow a monotonic trend. Although BMI appears to increase monotonically starting from a few months before the separation, the deviations in BMI between children experiencing separation and the profile of children from intact families are not statistically significant until 24 months after the separation. Differences in overweight/obesity probability are not statistically significant until 36 months after the separation. The effect of parental separation on children’s BMI becomes increasingly important and statistically significant two to three years after separation, which could be the consequence of a cumulative penalty over time. The finding that it takes longer for the risk of overweight/obesity to show significant differences echoes the indication that continuous increases in BMI result in increases in the risk of becoming overweight/obese later on.^15^

These results underscore the importance of treating parental separation as a process over a sufficiently long period and indicate that a model such as Model 1, which does not allow for flexible timing of the effect, would lead us to overestimate the short-run impact of separation and underestimate its long-run impact by about 50 %.

Table 5 expands on the results shown in Table 4 by showing whether the negative effect of separation differs based on whether a child experienced the separation before or after age 6. For all three outcome measures, the effect is stronger for children whose parents separated before they turned 6. This pattern is illustrated in Figs. 3, 4 and 5. The pattern for children who experienced a parental separation before age 6 is clear and monotonic; that is, the association between parental separation and children’s weight gain is significant starting from 12 months after the separation. In contrast, for the results for the children who experienced parental separation after age 6, we find significant differences only in the 24–36 months following the separation. The fact that we do not observe the same results 36 months after separation could be due to the small number of children who experienced separation after age 6 and whom we observe until 36 months following separation. The results for overweight/obesity do not differ significantly depending on whether the child experienced parental separation before or after age 6. This could be attributable to imprecise estimation of the parameters because we split the sample into two smaller groups and because the trend is nevertheless positive. It is also possible that the age-at-separation effect is confounded with a period effect. According to the resources arguments, the effect of separation might be stronger in the aftermath of an economic recession (that is, for the separations after the children in our sample reached age 6, around 2007). Given that our results point in the opposite direction, it appears that the period is not the main driver of the age-at-separation pattern.

We ran a series of sensitivity analyses to test the robustness of our results. First, we ran the models presented in Table 4 on an unbalanced sample. The results, presented in Table A2 of the online appendix, were virtually identical to those presented in Table 4. This is unsurprising because the unbalanced sample is only marginally larger than the analytical sample used in the analyses and because the fixed-effects model is estimated from observations that were observed at least twice. Second, we looked at whether the results were similar if we stratified the analyses for boys and girls. The results (available upon request) showed that the overall pattern of a strengthening association between parental separation and children’s health with the length of time since separation is observed for both boys and girls. Third, we estimated the models excluding families who experienced multiple separations (Figs. A3–A5, online appendix). As noted in the data section, children who experienced coresidence with a stepparent and another separation (75 cases) are included in the analytical sample because repartnering and complex family trajectories are part of the separation process and are among the mechanisms that could link parental separation to children’s outcomes. Nonetheless, when we excluded these children, the results were unchanged, which suggests that the patterns we observe are not driven by this group of children. Finally, as an additional test that the results are not driven by the relationship between age and body weight, we also estimated models using within-sample rank-based measures of BMI as the dependent variable. Such measures are fully independent with respect to children’s age and gender and therefore show no association with age. Again, the results (available on request) exhibited the same patterns as our main BMI *z* score regressions.

## Discussion

In this article, we investigate the relationship between parental separation and children’s BMI and overweight/obesity risk in the UK. We treat parental separation as a process, and we analyze variations in children’s physical health before and after the date of their parents’ separation. To account for the potential correlation between children’s physical health and unobserved factors associated with parental separation, such as socioeconomic background and other time-invariant parental characteristics, we estimate fixed-effects models. The results show little evidence of anticipation effects in the buildup to parental separation or of any large, statistically significant change in children’s physical health immediately after separation. However, our results suggest that the effect gradually accumulates and results in a longer-term effect of parental separation on children’s health.

Are our estimates of the effect of separation on child health large? Childhood obesity and overweight are important policy concerns in most advanced economies and especially in the United States and the United Kingdom, where the prevalence of childhood overweight/obesity is high (National Obesity Forum 2014). A wide range of policy interventions have been applied in various settings, such as school-, home-, or community-based interventions to prevent normal weight children from becoming overweight or obese (Wang et al. 2013). Two recent meta-analyses of such programs (i.e., Stice et al. 2006; Wang et al. 2013) implemented in high-income countries reported that more than one-half of these prevention programs had no statistically significant effects. The average sizes of the effects of those that reported statistically significant effects are, from a clinical perspective, *medium* (i.e., *r* = .22 (*p* < .001); Stice et al. 2006) or *moderate* (i.e., ~0.30 mean difference in BMI; Wang et al. 2013). These effect sizes are smaller than (or comparable to) the effect sizes we find in our analyses, especially when we look at the results over the longer term and as the time since separation increases.

Our results help to answer the questions of *whether* and *how much* separation affects children’s physical health but not the question of *why*. Identifying the specific mechanisms that drive the impact of separation on children’s physical health is beyond the scope of our analysis. The MCS provides a limited set of variables that we can use to test the mechanisms through which parental separation affects children’s health. Nonetheless, we explore a few of the potential pathways by including variables in our models that might capture some of the mechanisms discussed in the Background section (i.e., resources, health behaviors, parental well-being).

First, we run a supplementary set of models that include as additional covariates four time-varying variables factors that are observed continuously between Waves 1 and 5: (1) family income quintile (adjusted for family size), (2) depression in the main parent, (3) number of siblings, and (4) the presence of a stepparent. We assess whether and, if so, how the separation effects are attenuated after their inclusion in the models. The results, shown in Table A3 (online appendix), indicate that including these variables does not attenuate the association between parental separation and an increase in children’s BMI and risk of overweight/obesity—suggesting that they do not represent the main mechanisms. Because of missingness on the additional control variables, the sample size used to estimate these models is smaller than the one used in Table 4. As a robustness check, we replicate the results presented in Tables 4 using this smaller sample, and the results (not shown) are qualitatively similar.

Second, we run a set of models including (in addition to the four variables used in Table A4 of the online appendix) additional variables only observed from Wave 3 onward (i.e., whether the child has breakfast every day, eats fruit every day, watches TV for three or more hours every day, engages in active playing, and has a regular bedtime) that have been identified in the previous literature as relevant factors in a child’s risk of having a high BMI or of being overweight/obese (e.g., Goisis et al. 2016; Hancox et al. 2004; Schmeer 2012). The first two specifications, shown in Table A4 (online appendix), reveal that when we exclude Wave 2, the overall results are qualitatively similar to the main results presented in Table 4. The third specification shows that the adjustment for all the control variables attenuates the coefficient very marginally.

Third, although our analyses suggest that these potential pathways cannot fully explain the link between parental separation and children’s physical health, we run a further assessment to determine whether any of these pathway variables change because of parental separation. We do so by running fixed-effects models using each of the potential pathways just described as a dependent variable. The results, presented in Table A5 (online appendix), show that as expected, many of these variables respond to parental separation: that is, after parental separation, children’s health behavior is more likely to deteriorate (except that they are more likely to eat fruit every day), the household is more likely to be in the bottom income quintile, and the main parent is more likely to be depressed. Moreover, some of these effects appear to strengthen with the time since the separation. Because these resource and behavior variables respond to separation and affect children’s risk of having high BMI or overweight/obesity, it is somewhat surprising that these factors do not help to explain the link between parental separation and children’s physical health. One possible explanation is that these indicators are rather crudely measured (i.e., binary variables) and thus fail to provide precise information about children’s energy consumption and expenditures. Future research should seek to replicate these analyses using data sets with more detailed and frequently collected information on children’s diets and activities.

## Conclusions

Many studies have shown that parental separation is negatively associated with children’s cognitive skills, educational outcomes, and emotional and psychological well-being; see the recent literature reviews by Amato (2010), Bernardi et al. (2013), and McLanahan et al. (2013). Fewer studies—all of which focused on the United States, and most of which used cross-sectional data—have examined the association between family breakup and children’s physical health (e.g., Bzostek and Beck 2011; Chen and Escarce 2010; McConley et al. 2011; Schmeer 2012; Yannakoulia et al. 2008). This is a serious gap in the literature given that parental separation could negatively affect children’s physical development and have long-lasting consequences on their health. We address this gap using longitudinal data from the United Kingdom and carefully investigate whether parental separation is associated with children’s physical health by controlling for unobserved time-invariant parental characteristics (which might be associated both with the risk of separation and children’s health) and looking at the effects both before and after separation.

Our results show that parental separation is associated with increases in children’s BMI and their risk of overweight/obesity. In the pre-separation and short-term after separation, the association between parental separation and children’s BMI and the risk of overweight/obesity is negligible both statistically and substantively. We instead find a longer-term effect as the association between parental separation and children’s BMI and the risk of child overweight/obesity gets stronger, both statistically and substantively, over time.

Some limitations of our findings should be borne in mind. First, for space and scope reasons, we do not investigate whether multiple family transitions and complex family trajectories mediate the effects of parental separation on the BMI for the subset of children who experience them. Although our sample does not contain many of these children and excluding them does not affect our findings, the associations between different stages of parental separation (pre- and post-separation effects) and physical health for those children might be very different. Future research, using different data with larger sample sizes, should examine family complexity more in detail. More generally, more detailed data on health behaviors and food consumption, such as in the form of children’s food and time diaries linked to the panel data, which would document changes in net nutrition, could help future research examine the mechanisms at play in greater detail. Second, to identify a distinct effect of time since separation for younger and older children with our cohort data, we have to assume away any specific time effects, an assumption that cannot be tested. Finally, because of attrition, our results likely underestimate the full effect of separation on health, at least to the extent that the most disruptive separations are both more likely to lead to sample drop out and larger effects on BMI.

Notwithstanding these limitations, our results underscore the importance of modelling parental separation as a process with potentially long-lasting consequences. They also suggest that looking at the average effect might lead us to underestimate the association between parental separation and children’s weight trajectories because the magnitude of this association becomes stronger as the time since separation increases. A key strength of our analysis is that the results were consistent when we use age- and gender-adjusted BMI measures, which effectively remove biological changes in BMI as children grow older. This enables us to argue that the observed effects are not merely capturing an “age effect” or the fact that BMI and the risk of overweight/obesity increase with age but instead reflect the effect of parental separation on children’s health above and beyond a common BMI-age profile.

Our findings are consistent with the evidence provided by Pronzato and Aassve (2019), showing that parental separation has a longer-term effect on children’s behavioral outcomes. Also, we find that the effect is especially strong among children whose parents separated before they turned 6. This finding is in line with the strand of literature on children’s well-being suggesting that parental separation is more detrimental if the child is young (Amato 2001; King 2002). Because the association between parental separation and children’s weight gain strengthens as the time since the separation increases, efforts to prevent these children from gaining weight should start early, and soon after separation. Intervening early could help to prevent—or at least attenuate—the process that leads some children to develop unhealthy adiposity trajectories.

## Electronic supplementary material

ESM 1 (PDF 3897 kb)

## Footnotes

1 Two recent studies analyzed the effects of childhood family structure changes on *adult* physical health using data sets from the United States: Hernandez et al. (2014) focused on adult obesity and overweight using NLSY data, and Slade et al. (2017) focused on young adults’ health behaviors using the National Longitudinal Study of Adolescent Health. A few other recent (cross-sectional) studies have compared the physical health of children living in different family structures (e.g., with a single mother or in a reconstituted family). See, for example, Pearce et al. (2015), Parikka et al. (2015), and Hohwu et al. (2015). These studies did not focus on parental separation or children whose parents separated after they were born.

2 This theory suggests that children who experience parental separation might be worse off than children who grew up in stable single-parent households given that the latter do not experience adjustment to the new environment and stress induced by the change. See Mariani et al. (2017) for a discussion about the United Kingdom.

3 Gambaro et al. (2017) reported that 40 % of the children in the MCS experienced a residential change before age 5, and more than 60 % of these children experienced an improvement in their residential environment (e.g., a larger house or a better neighborhood). The authors also found an association with parental separation and residential mobility.

4 We also exclude two observations with implausible BMI values (>60) and 41 cases in which the biological parents separated after Wave 1 but reconciled before the Wave 5 interview.

5 Many (80 %) missing cases are lost to follow-up. The remaining 20 % are largely cases with missing BMI at all waves.

6 For completeness, we repeat our estimation on the unbalanced sample of children who were observed at least at Waves 1 and 2—that is, the children who contributed at least two observations of child health and time to separation. This provides us with a larger sample of 7,765 children. This unbalanced sample may be less sensitive to attrition bias. However, for two reasons, we do not consider it as our baseline sample. First, given the cohort study design, the unbalanced sample overrepresents observations at younger ages, and the adjustment of the design weights to account for attrition (even if it is reduced) is open to question. Second, the unbalanced design limits the options to model the process of separation and its impact on child health.

7 We use the user-written Stata routine *zanthro* for this standardization (Cole et al. 2005).

8 In addition to the two approaches described in this section, we experimented with several alternative regression-based standardizations by age and gender, using ordinary least squares (OLS) and median regressions applied to the MCS data. Most importantly, we examined purely rank-based measures that eliminate any age- and gender-associated variation in BMI. They were constructed by looking at the rank of children’s BMI in our sample at each age by gender. Rank-based measures were insensitive to variations in BMI distributions as children age. The results of the models using these alternative standardization methods produced qualitatively similar results (not reported but available upon request).

9 The sample of children whose parents separated includes some with more complex family trajectories (i.e., the resident parent repartnered or had multiple separations). The event of interest here is the first separation of the child’s biological parents. We exclude children whose biological parents separated and then reconciled. We do not, however, exclude those who lived with a stepparent who then separate from the biological parent (i.e., children who experience multiple separations (75 cases)) because such experiences are part of the separation process and are among the mechanisms that may link parental separation to children’s outcomes.

10 Manning and Smock (2005) cautioned about potential measurement error regarding the timing of union *formation* among cohabiting couples. We expect *dissolution* to be recalled more accurately because, *de facto*, it happens in the more recent past of respondents. Also, we do not expect systematic measurement error around the dissolution dates. Nonetheless, as a check, we replicated our main regression analysis after excluding cohabiting couples (among which measurement error is more likely) and obtained results very similar to those reported in the article.

11 For the two cases in which information on the year but not the month of separation is available, we impute the separation as having occurred in June. For cases with missing information on the month/year of separation (94 cases) but with complete information on partnership status and all other variables necessary in this analysis, we impute the separation as having occurred at the midpoint between the interview dates before and after separation.

12 For our standardized BMI measures, the slope coefficients θ_1_ and θ_2_ are 0 in the population because the effects of age (and gender) have been purged from the measure by construction. However, because we estimate the models in our balanced sample of children observed in all four MCS waves of interest, we address potential age-related differential attrition while keeping the quadratic form for *f* in the regression model.

13 Allowing for gender-specific parameters in the age-BMI functional relationship does not change our estimates of the effect of separation. We therefore stick to a more parsimonious specification common to both sexes.

14 Children who experienced parental separation within 12 months after their Wave 5 interview are not coded as being in the build-up phase because we use information only until the Wave 5 interview. We do not expect this to be of major concern because we find no anticipation effect on children’s BMI with the Wave 1–5 data.

15 As a robustness check, we replicate the models on obesity/overweight running a fixed effects logit model. The results (available upon request) show a consistent picture.
