# Supplementary material for: Do Children Carry the Weight of Divorce?
Source: Demography. 2019 Jun 11;56(3):785–811. doi: 10.1007/s13524-019-00784-4 (PMC6592970; doi:10.1007/s13524-019-00784-4)

Online Appendix

Table A1. Descriptive characteristics of the analytical sample

	Wave 2	Wave 3	Wave 4	Wave 5
Cohort member age in days (mean)	1139	1903	2637	4076
Min age in days	1023	1614	2326	3713
Max age in days	1631	2238	2941	4474
Number of observations		7,574		
**Family characteristics**				
Top income quintile (%)	10.6	10.6	11.7	9.6
Bottom income quintile (%)	23.7	23.7	24.0	24.4
Number of biological and step-siblings (mean)	1.2	1.4	1.5	1.5
Presence of a step-parent in the household (%)	0.7	2.0	1.6	5.4
Main respondent is depressed (%)	30.6	27.0	29.5	39.4
Number of observations*		7,052		

*Note: The number of observations drops because of some cases with missing values on the additional family covariates.

Table A2. Fixed-effects regression model on BMI, BMI-z scores, and overweight on the unbalanced sample

	BMI		BMI z-scores		Overweight	
	**Model 1**	**Model 2**	**Model 1**	**Model 2**	**Model 1**	**Model 1**
Cohort member age in days	-0.003***	-0.003***	-0.000***	-0.000***	-0.000***	-0.000***
	(0.000)	(0.000)	(0.000)	(0.000)	(0.000)	(0.000)
Cohort member age in days^2	0.000***	0.000***	0.000***	0.000***	0.000***	0.000***
	(0.000)	(0.000)	(0.000)	(0.000)	(0.000)	(0.000)
Parents separated	0.291***		0.086***		0.022	
	(0.077)		(0.029)		(0.014)	
Reference Category: At least 12 months prior to separation		(dropped)		(dropped)		(dropped)
Pre-sep 0-12 months						
		0.108		0.044		-0.003
Post-sep 0-11months		(0.088)		(0.042)		(0.020)
		0.205*		0.057		0.027
Post-sep 12-23months		(0.114)		(0.054)		(0.017)
		0.264**		0.094**		0.005
Post-sep 24-35months		(0.111)		(0.047)		(0.019)
		0.426***		0.129***		0.034
Post-sep 36+months		(0.129)		(0.045)		(0.023)
		0.443***		0.127***		0.036*
Constant		(0.121)		(0.045)		(0.019)
	19.010***	19.021***	0.837***	0.839***	0.320***	0.321***
Number of observations	7,765		7,765		7,765	
Number of groups	31,018		31,018		31,018	

Note: Standard errors are provided in parenthesis *** p<0.01, ** p<0.05, * p<0.1. Standard errors clustered at the primary sampling unit.

Table A3. Fixed-effects regression model on BMI, BMI-z scores, and overweight with adjustment for time-varying family characteristics

	BMI		BMI z-scores		Overweight	
	**Model 1**	**Model 2**	**Model 1**	**Model 2**	**Model 1**	**Model 1**
Cohort member age in days	-0.003***	-0.003***	-0.000***	-0.000***	-0.000***	-0.000***
	(0.000)	(0.000)	(0.000)	(0.000)	(0.000)	(0.000)
Cohort member age in days squared	0.000***	0.000***	0.000***	0.000***	0.000***	0.000***
	(0.000)	(0.000)	(0.000)	(0.000)	(0.000)	(0.000)
Parents separated	0.264***		0.070**		0.020	
	(0.085)		(0.032)		(0.015)	
Reference Category: At least 12 months prior to separation						
Pre-sep 0-12 months		0.090		0.038		-0.003
		(0.096)		(0.045)		(0.021)
Post-sep 0-11months		0.150		0.037		0.024
		(0.120)		(0.058)		(0.019)
Post-sep 12-23months		0.214*		0.069		-0.002
		(0.125)		(0.052)		(0.020)
Post-sep 24-35months		0.439***		0.128***		0.037
		(0.136)		(0.048)		(0.025)
Post-sep 36+months		0.444***		0.115**		0.039*
		(0.137)		(0.050)		(0.021)
Main respondent is depressed	0.112***	0.113***	0.026*	0.027*	0.007	0.008
	(0.038)	(0.038)	(0.016)	(0.016)	(0.007)	(0.007)
Bottom income quintile (reference top income quintile)	-0.015	0.005	-0.013	-0.010	0.001	0.004
	(0.097)	(0.098)	(0.034)	(0.034)	(0.016)	(0.017)
Second income quintile	0.038	0.047	0.001	0.003	-0.004	-0.002
	(0.073)	(0.073)	(0.029)	(0.029)	(0.013)	(0.013)
Third income quintile	0.112*	0.117**	0.037	0.039*	0.011	0.012
	(0.060)	(0.060)	(0.023)	(0.023)	(0.011)	(0.011)
Fourth income quintile	-0.010	-0.008	-0.002	-0.002	0.015*	0.015*
	(0.047)	(0.047)	(0.019)	(0.019)	(0.008)	(0.008)
Number of siblings in the household	-0.264***	-0.263***	-0.082***	-0.082***	-0.015***	-0.015***
	(0.040)	(0.040)	(0.015)	(0.015)	(0.006)	(0.006)
Presence of a step parent in the household	-0.074	-0.152	-0.011	-0.028	-0.007	-0.016
	(0.163)	(0.170)	(0.061)	(0.062)	(0.023)	(0.024)
Constant	19.132***	19.141***	0.884***	0.886***	0.325***	0.326***
	(0.086)	(0.085)	(0.039)	(0.039)	(0.016)	(0.016)
Number of observations	28,208		28,208		28,208	
Number of groups	7,052		7,052		7,052	

Note: Standard errors are provided in parenthesis *** p<0.01, ** p<0.05, * p<0.1. Standard errors clustered at the primary sampling unit.

Table A4. Fixed-effects regression model on BMI, BMI-z scores, and overweight for waves 3-5 only

	BMI			BMI z-scores			Overweight		
	Model 1	Model 2	Model 3	Model 1	Model 2	Model 3	Model 1	Model 2	Model 3
Cohort member age in days	-0.003***	-0.003***	-0.002***	-0.001***	-0.001***	-0.001***	-0.000***	-0.000***	-0.000***
	(0.000)	(0.000)	(0.000)	(0.000)	(0.000)	(0.000)	(0.000)	(0.000)	(0.000)
Cohort member age in days squared	0.000***	0.000***	0.000***	0.000***	0.000***	0.000***	0.000***	0.000***	0.000***
	(0.000)	(0.000)	(0.000)	(0.000)	(0.000)	(0.000)	(0.000)	(0.000)	(0.000)
Parents separated	0.363***			0.114***			0.026		
	(0.099)			(0.038)			(0.017)		
Reference Category: At least 12 months prior to separationpre		(dropped)	(dropped)		(dropped)	(dropped)		(dropped)	(dropped)
Pre-sep 0-12 months		0.202	0.166		0.078	0.067		-0.004	-0.009
		(0.140)	(0.137)		(0.056)	(0.056)		(0.023)	(0.023)
Post-sep 0-11months		0.156	0.108		0.062	0.046		0.019	0.014
		(0.153)	(0.156)		(0.075)	(0.077)		(0.024)	(0.024)
Post-sep 12-23months		0.391***	0.351**		0.160**	0.146**		0.021	0.016
		(0.144)	(0.149)		(0.063)	(0.067)		(0.024)	(0.023)
Post-sep 24-35months		0.496***	0.476***		0.146***	0.137***		0.020	0.015
		(0.164)	(0.162)		(0.051)	(0.052)		(0.026)	(0.026)
Post-sep 36+months		0.613***	0.598***		0.188***	0.180***		0.047*	0.040
		(0.165)	(0.169)		(0.062)	(0.064)		(0.025)	(0.025)
Regular breakfast (no/yes)			0.060			-0.004			-0.019
			(0.079)			(0.031)			(0.012)
Eat fruit daily (no/yes)			0.159***			0.058***			0.026***
			(0.047)			(0.018)			(0.007)
TV more than 3hrs/day (no/yes)			0.150**			0.054**			0.021**
			(0.064)			(0.023)			(0.009)
Regular active play (no/yes)			0.019			-0.012			-0.011
			(0.046)			(0.019)			(0.008)
Regular bedtime (no/yes)			0.033			0.043			-0.007
			(0.080)			(0.027)			(0.011)
Main respondent depressed (no/yes)			0.121**			0.014			-0.001
			(0.047)			(0.019)			(0.008)
Bottom income quintile (ref: top quintile)			-0.026			-0.017			-0.005
			(0.095)			(0.034)			(0.017)
Second income quintile			0.045			0.007			-0.014
			(0.069)			(0.027)			(0.013)
Third income quintile			0.116**			0.037*			0.001
			(0.056)			(0.021)			(0.011)
Fourth income quintile			0.015			0.011			0.012
			(0.045)			(0.017)			(0.008)
Number of siblings in the household			-0.271***			-0.079***			-0.017**
			(0.051)			(0.017)			(0.008)
Presence of a step-parent in the household			-0.130			-0.038			-0.006
			(0.184)			(0.069)			(0.025)
Constant	18.727***	18.756***	18.736***	1.153***	1.162***	1.184***	0.349***	0.351***	0.376***
Number of observations		21,075			21,075			21,075	
Number of groups		7,052			7,052			7,052	

Note: Standard errors are provided in parenthesis *** p<0.01, ** p<0.05, * p<0.1. Standard errors clustered at the primary sampling unit.

Table A5. Fixed-effects regression model on the control variables for waves 3-5

	CM has breakfast every day		CM eats fruit daily		CM watches TV 3+ hours every day		CM does regular active playing		CM has no regular bedtime		Main respondent depressed		Family in the bottom income quintile		Presence of a step-parent in the hh	
	**Model 1**	**Model 2**	**Model 1**	**Model 2**	**Model 1**	**Model 2**	**Model 1**	**Model 2**	**Model 1**	**Model 2**	**Model 1**	**Model 2**	**Model 1**	**Model 2**	**Model 1**	**Model 2**
Child age in days	0.000***	0.000***	0.000***	0.000***	0.000	0.000	0.000	0.000	-0.000	-0.000	-0.000	-0.000	0.000*	0.000**	-0.000***	-0.000***
	(0.000)	(0.000)	(0.000)	(0.000)	(0.000)	(0.000)	(0.000)	(0.000)	(0.000)	(0.000)	(0.000)	(0.000)	(0.000)	(0.000)	(0.000)	(0.000)
Child age in days squared	-0.000***	-0.000***	-0.000***	-0.000***	-0.000	-0.000	-0.000***	-0.000***	0.000	0.000	0.000***	0.000***	-0.000**	-0.000**	0.000***	0.000***
	(0.000)	(0.000)	(0.000)	(0.000)	(0.000)	(0.000)	(0.000)	(0.000)	(0.000)	(0.000)	(0.000)	(0.000)	(0.000)	(0.000)	(0.000)	(0.000)
Parents separated	-0.031*		0.074***		-0.009		-0.222***		-0.007		0.059**		0.084***		0.133***	
	(0.018)		(0.026)		(0.017)		(0.023)		(0.015)		(0.025)		(0.017)		(0.013)	
Reference Category: At least 12 months prior to separation		(dropped)		(dropped)		(dropped)		(dropped)		(dropped)		(dropped)		(dropped)		(dropped)
Pre-sep 0-12 months		-0.018		0.106***		-0.013		-0.017		0.002		0.082*		-0.032		0.027
		(0.022)		(0.035)		(0.030)		(0.036)		(0.021)		(0.042)		(0.024)		(0.017)
Post-sep 0-11months		-0.011		0.060		0.016		-0.233***		0.010		0.133***		0.119***		0.032**
		(0.026)		(0.037)		(0.029)		(0.037)		(0.023)		(0.040)		(0.026)		(0.014)
Post-sep 12-23months		-0.060**		0.114***		-0.017		-0.217***		0.014		0.082**		0.123***		0.114***
		(0.027)		(0.041)		(0.028)		(0.033)		(0.021)		(0.036)		(0.028)		(0.024)
Post-sep 24-35months		-0.024		0.045		0.008		-0.259***		-0.026		0.049		0.071***		0.137***
		(0.025)		(0.041)		(0.024)		(0.037)		(0.023)		(0.038)		(0.027)		(0.025)
Post-sep 36+months		-0.043*		0.178***		-0.044*		-0.236***		-0.033		0.069*		-0.012		0.255***
		(0.022)		(0.034)		(0.023)		(0.030)		(0.020)		(0.037)		(0.023)		(0.021)
Number of observations	21,075		21,075		21,075		21,075		21,075		21,075		21,075		21,075	
Number of groups	7,052		7,052		7,052		7,052		7,052		7,052		7,052		7,052	

Note: Standard errors are provided in parenthesis *** p<0.01, ** p<0.05, * p<0.1. Standard errors clustered at the primary sampling unit.

Figure A1. Evolution of BMI distribution over age, by sex

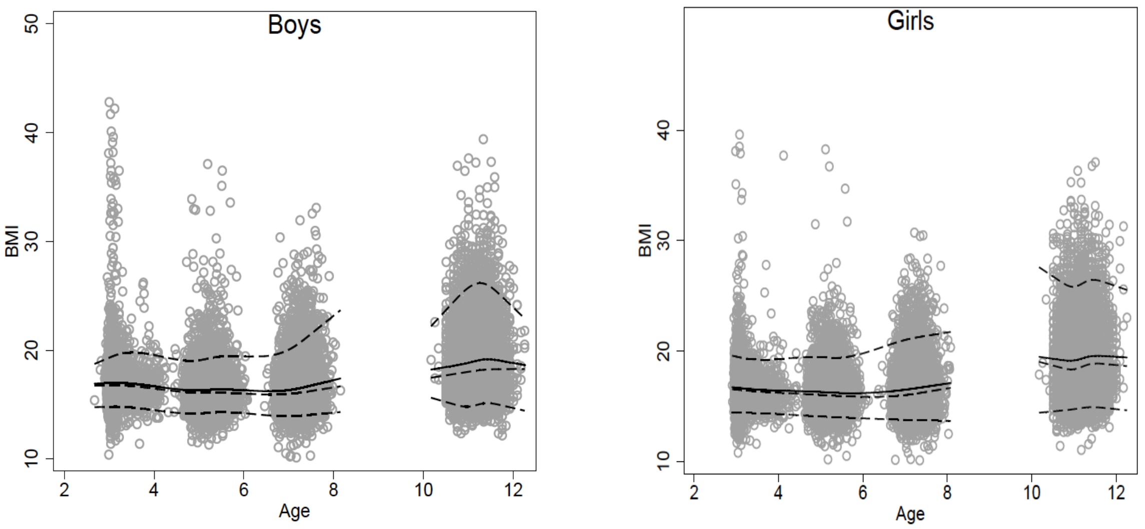

Note: Data from all children with BMI measurements in any Millennium Cohort Study (MCS) wave (full MCS sample). The four clouds of points on each figure correspond to the four MCS waves 2-5. Age is measured in days, since the day of birth divided by 365. Average BMI is indicated by the solid line. Estimates of the 5th, 50th, and 95th percentiles of the BMI distribution are shown by the dashed lines.

Figure A2. Evolution of BMI z-scores over age, by sex

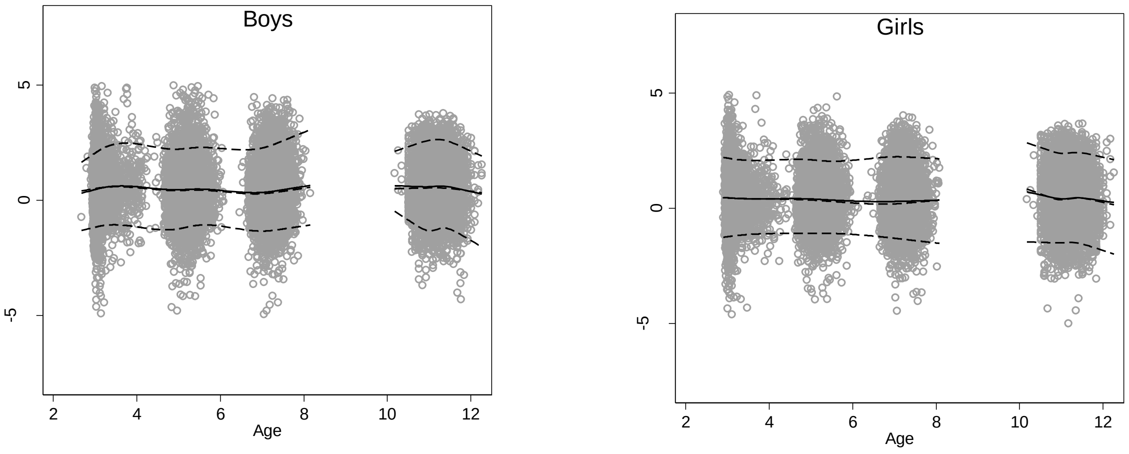

Figure A3. Predicted values of BMI for the time to/from separation excluding multiple separations

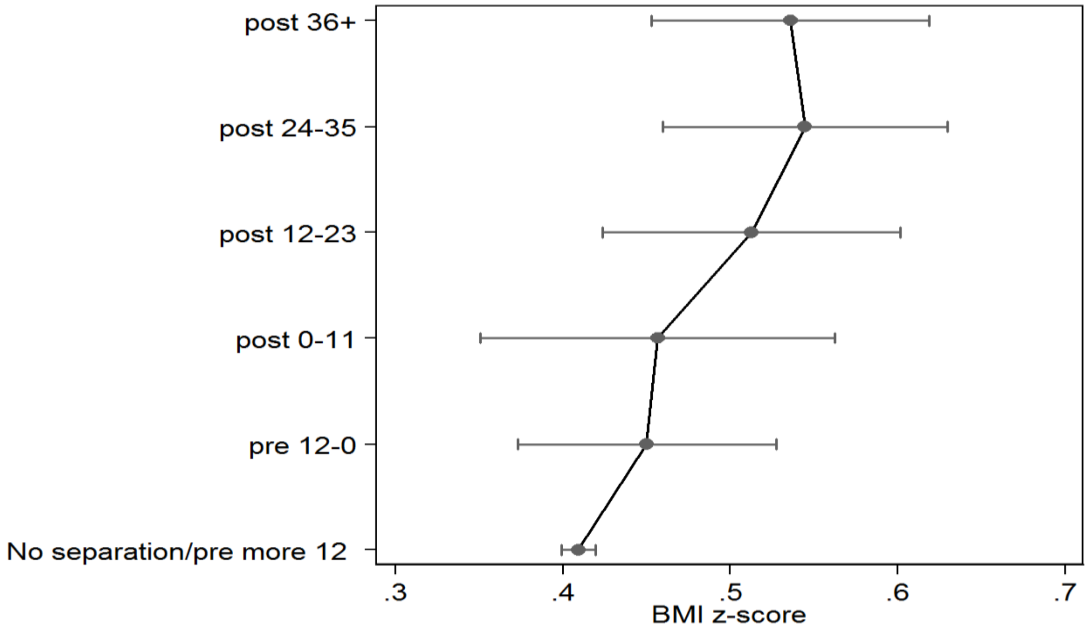

Figure A4. Predicted values of BMI for the time to/from separation excluding multiple separations

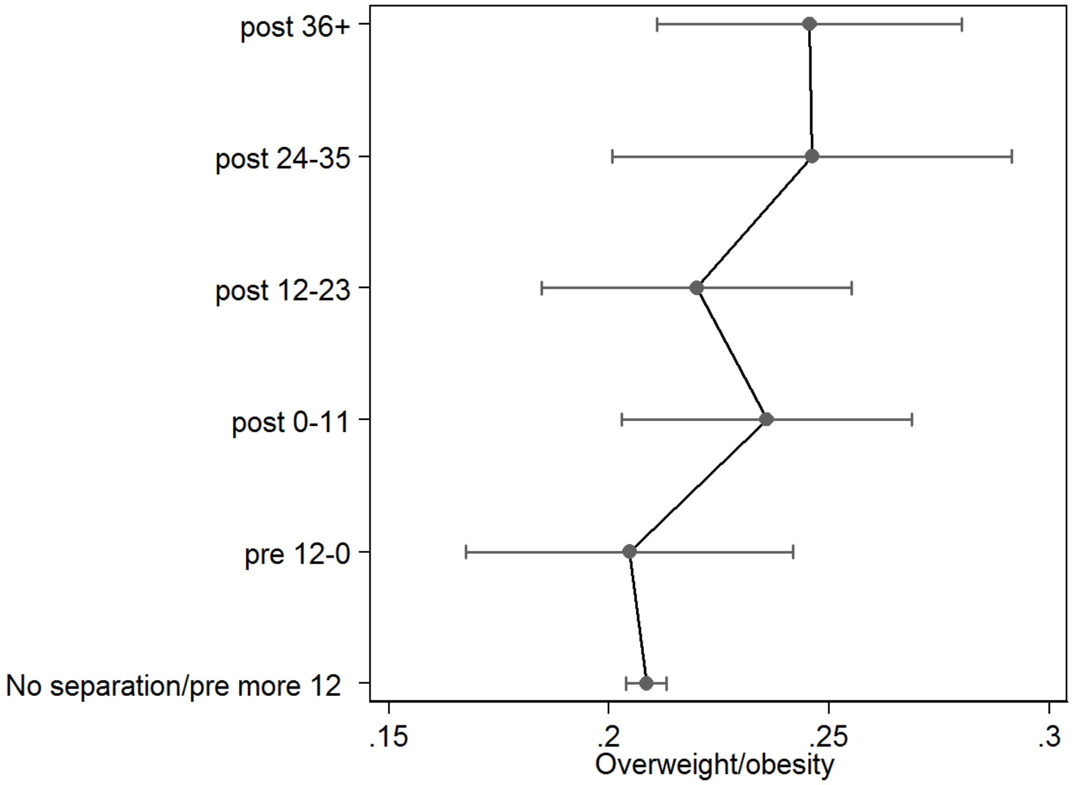

Figure A5. Predicted values of overweight for the time to/from separation excluding multiple separations

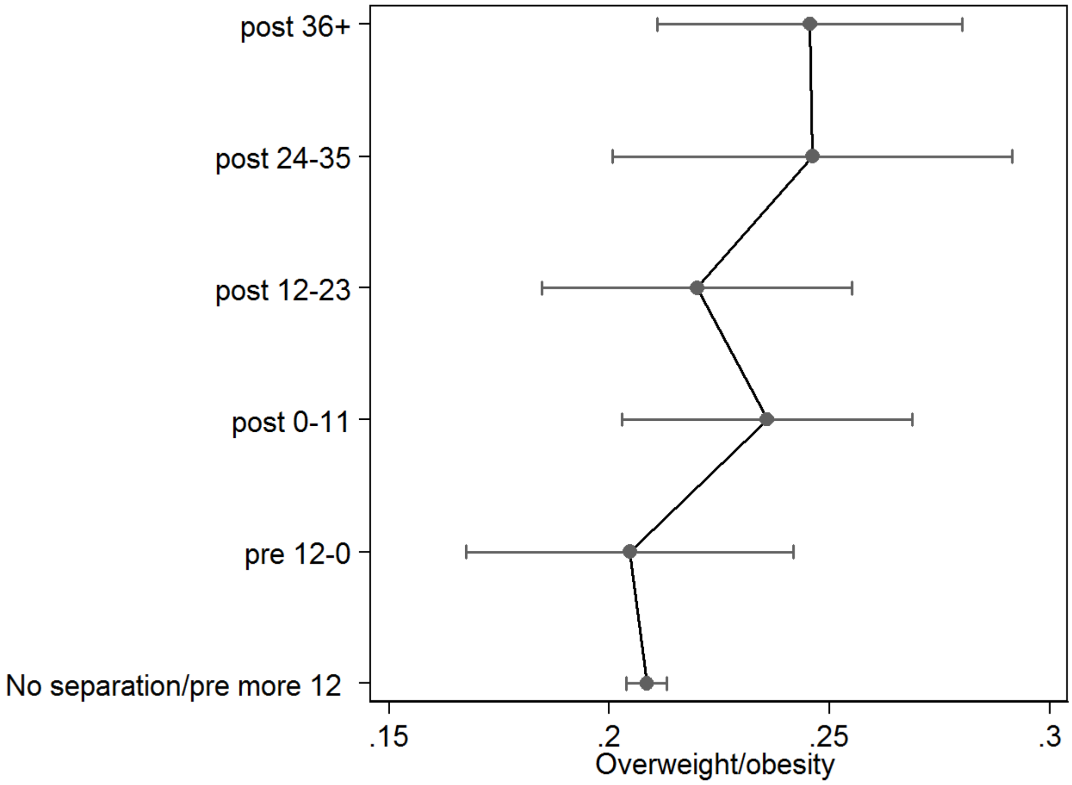

Supplement: Supplementary file 1 — (PDF 3897 kb) [file 13524_2019_784_MOESM1_ESM.pdf]
